# Supplementary material for: JUN upregulation drives aberrant transposable element mobilization, associated innate immune response, and impaired neurogenesis in Alzheimer’s disease
Source: Nat Commun. 2023 Dec 4;14:8021. doi: 10.1038/s41467-023-43728-8 (PMC10696058; doi:10.1038/s41467-023-43728-8)
Supplement: Supplementary file 1 — Supplementary Information [file 41467_2023_43728_MOESM1_ESM.pdf]

Supplementary Figures

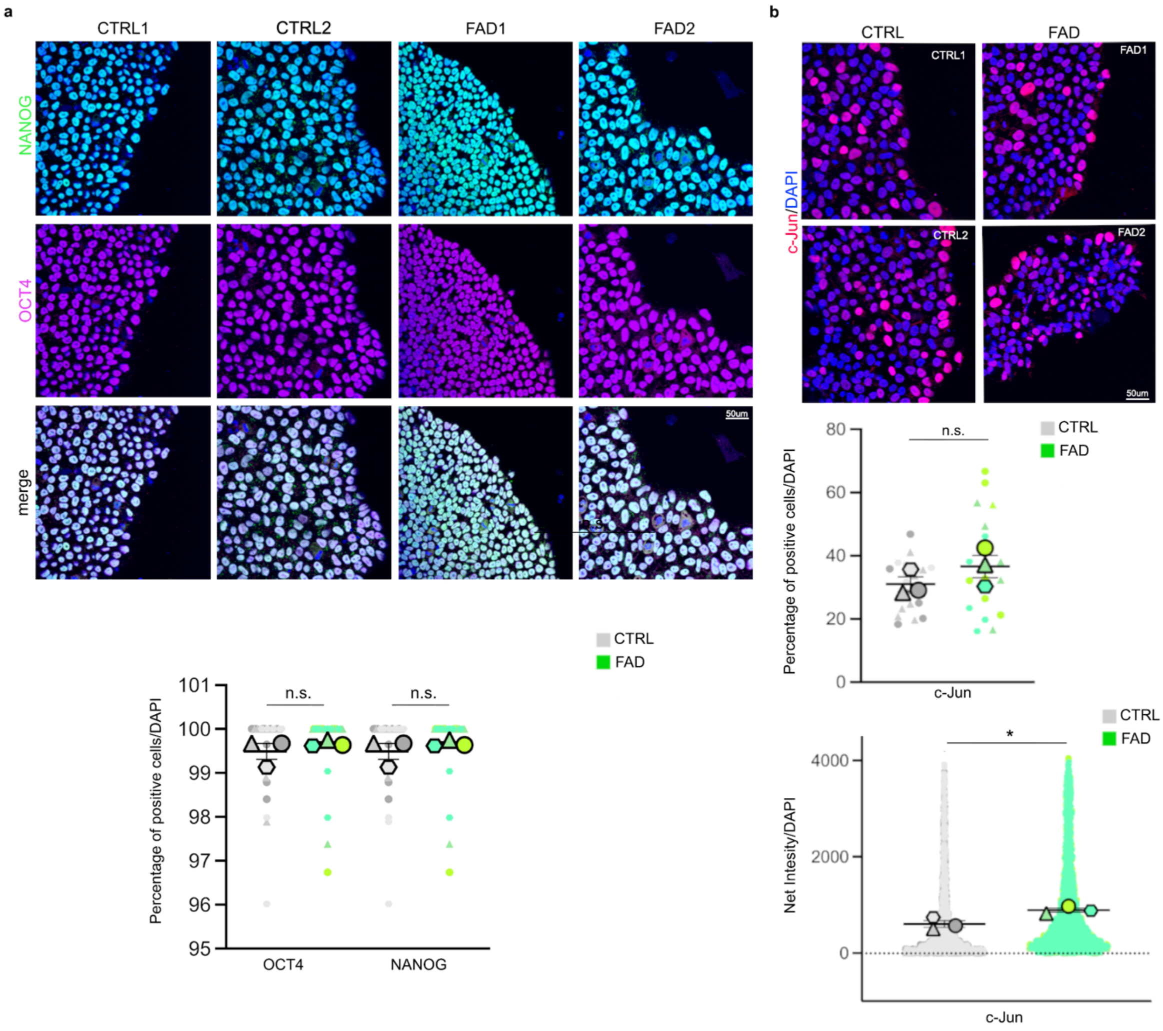

Supplementary Figure 1. **Pluripotency and JUN expression profiles of CTRL and FAD-derived iPSCs**. **a.** and **b**. Immunofluorescence quantifying the expression of **a**. pluripotency markers OCT4 (purple) and NANOG (green) and **b**. endogenous c-Jun in iPSCs derived from CTRLs and FAD. The quantification of immunofluorescence in panel b is reported as the percentage of expressing cells (upper superplot) and expression of net intensity (bottom superplot). Each IF was replicated three times with similar results (n= 3 independent experiments). The IF data are quantified using a Superplot, which concisely visualizes individual data points and their averages. The distinct combinations of colors and shapes indicate the three independent experiments performed. Each small dot in the graph corresponds to a specific data point representing an analyzed image or cells. The larger dots represent the average values calculated from the respective data points. A Repeated Measures ANOVA test was used for the comparison between two groups. A value of $P < 0.05$ was considered

significant; * P<0.05(c-Jun intensity P=0.0123); n.s., not significant (OCT4 P=0.609; NANOG P=0.609; c-Jun P=0.107). Error bars report the Standard Error

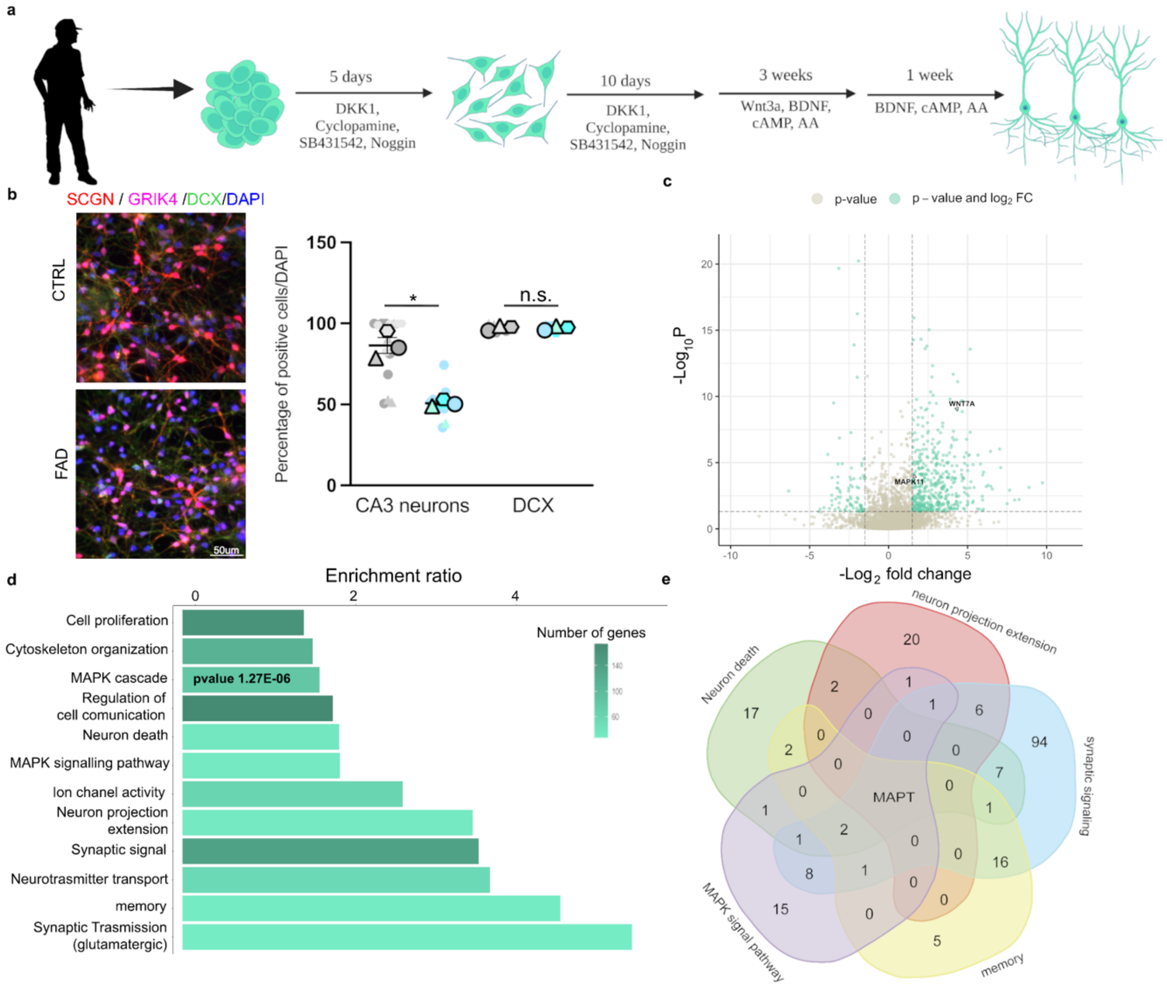

Supplementary Figure 2. **Dysregulated transcriptional networks and pathways in FAD CA3 hippocampal neurons**. **a**. A protocol scheme for CA3 hippocampal neuron differentiation (made with Biorender.com). **b**. Immunofluorescence for CA3 neuron markers. GRIK4 (pink) and SCGN (red) were expressed in CTRL and FAD neurons demonstrating their proper differentiation. The relative quantification shows that FAD hpNPCs failed to differentiate into CA3 neurons, despite the same percentage of DCX positive late progenitors (in green) in the culture. Scale bar 50um, 40X magnification. DAPI staining on nuclei in blue. Each IF was replicated three times with similar results (n= 3 independent experiments). The IF data are quantified using a Superplot, which concisely visualizes individual data points and their averages. The distinct combinations of colors and shapes indicate the three independent

experiments performed. Each small dot in the graph corresponds to a specific data point representing an analyzed image or cells. The larger dots represent the average values calculated from the respective data points. A Repeated Measures ANOVA test was used for the comparison between two groups. A value of $P < 0.05$ was considered significant; * $P<0.05$ (CA3 $P=0.0241$); n.s., not significant (DCX $P=0.94$). Error bars report the Standard Error **c**. Volcano plot displaying the 563 differentially expressed genes in FAD CA3 neurons relative to CTRL CA3 neurons. Teal = differentially expressed genes passing significance thresholds p-value < 0.05 and $\log_2$(fold-change) +/– 1.5; Gray = not significant. 5% False Discovery Rate (FDR) was used to correct for multiple testing. **d**. Enriched pathways associated with the 563 differentially expressed genes in FAD CA3 neurons predicted by WebGestalt. 5% False Discovery Rate (FDR) was used to correct for multiple testing. **e**. Venn diagram showing the genes shared across five enriched pathways in AD. (venn diagram was made using https://bioinformatics.psb.ugent.be).

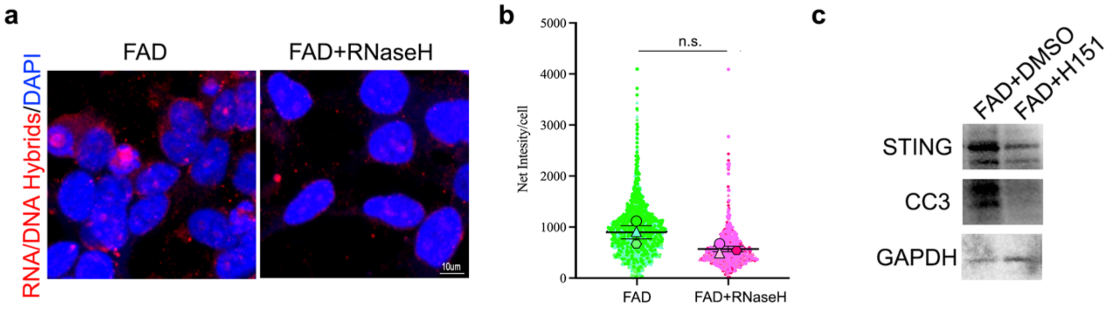

Supplementary Figure 3. **Validation of the RNA-DNA hybrids-STING-cell death axis.**

a, b. Immunofluorescence for RNA-DNA hybrids and relative quantification. RNA-DNA hybrids expression in FAD hpNPCs decreases upon treatment with RNaseH. Scale bar 10um, 60X magnification, 3x zoom. DAPI staining on nuclei in blue. Each IF was replicated three times with similar results (n= 3 independent experiments). The IF data are quantified using a Superplot, which concisely visualizes individual data points and their averages. The distinct combinations of colors and shapes indicate the three independent experiments performed. Each small dot in the graph corresponds to a specific data point representing an analyzed image or cells. The larger dots represent the average values calculated from the respective data points. A Repeated Measures ANOVA test was used for the comparison between two groups. A value of $P < 0.05$ was considered significant; * $P<0.05$ (RNA-DNA hybrids $P=0.0216$). Error bars

report the Standard Error **c**. Immunoblots for STING and cleaved caspase 3 (CC3) on FAD hpNPCs treated with H151 compound (STING inhibitor) relative to DMSO-treated FAD progenitors. Each blot was replicated three times with similar results.

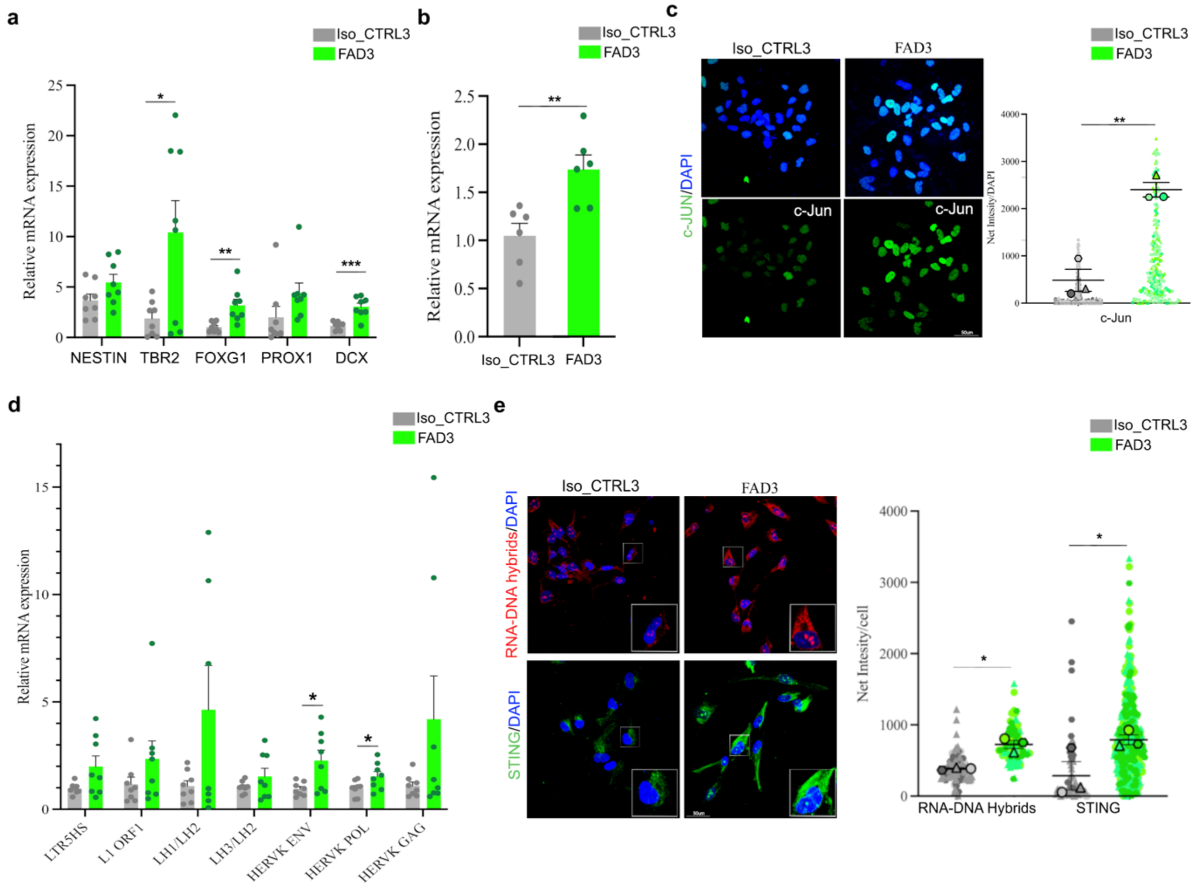

Supplementary Figure 4. **FAD isogenic iPSCs-derived hippocampal neural progenitors display impaired neurogenesis, JUN upregulation, and activation of the RNA-DNA hybrids-cGAS-STING axis**. **a**. qPCR analysis for hpNPC population markers. NESTIN – early precursors; TBR2/FOXG1 – intermediate progenitors; PROX1 – late progenitors; DCX – neuroblasts). Each dot in the bar plot represents an experiment (n= 8 independent experiments). A Two-sided T-test was used for the comparison between two groups. A value of $P < 0.05$ was considered significant; *P<0.05 (TBR2 P= 0.0117495); ** P<0.01 (FOXG1 P=0.003520) *** P<0.001 (DCX P=0.000234). Error bars report the Standard Error. **b**. qPCR for c-Jun in FAD3 and iso_CTRL. Each dot in the bar plot represents an experiment (n= 6 independent experiments). A Two-sided T-test was used for the comparison between two groups. A value of $P < 0.05$ was considered significant; ** P<0.01 (c-JUN P=0.0061). Error bars report the Standard Error. **c**.

Immunofluorescence for c-Jun and relative quantification. c-Jun is upregulated in FAD3 progenitors compared to their isogenic controls. Scale bar 50um, 60X magnification. DAPI staining on nuclei in blue. Each IF was replicated three times with similar results (n= 3 independent experiments). The IF data are quantified using a Superplot, which concisely visualizes individual data points and their averages. The distinct combinations of colors and shapes indicate the three independent experiments performed. Each small dot in the graph corresponds to a specific data point representing an analyzed image or cells. The larger dots represent the average values calculated from the respective data points. A Repeated Measures ANOVA test was used for the comparison between two groups. A value of $P < 0.05$ was considered significant; ** $P<0.01$ (c-Jun P=0.00145). Error bars report the Standard Error **d**. qPCR analysis for a group of TEs selected among those previously identified as aberrantly active in FAD hpNPC. Primers for HERVK/LTR5HS target individual ORFs from the LTR; Primers for L1-ORF1 target a conserved region in ORF1 of 6x; the other L1 primers target the L1PA2 family; the LH2/LH3 primers target the end of the 3' UTR; The LH1/LH2 primers target the 5' of the other amplicon. Each dot in the bar plot represents an experiment (n= 8 independent experiments). A Two-sided T-test was used for the comparison between two groups. A value of $P < 0.05$ was considered significant; *$P<0.05$ (HERK ENV P= 0.017304; HERK POL P= 0.045387); n.s., not signifucant (LTR5HS P=0.057709; L1 ORF1 P=0.207816; LH1/LH2 P=0.087310; LH2/LH3 P=0.220253; HERVK GAG P=0.145024). Error bars report the Standard Error. **e**. Immunofluorescence for RNA-DNA hybrids (red) and STING (green) and the relative quantification. FAD3 progenitors show RNA-DNA hybrid cytoplasmic accumulation and increased STING levels compared to their isogenic control. Scale bar 50um, 60X magnification. DAPI staining on nuclei in blue. White box in the bottom right corner is a 3x zoom of the boxed region in the panel. Each IF was replicated three times with similar results (n= 3 independent experiments). The IF data are quantified using a Superplot, which concisely visualizes individual data points and their averages. The distinct combinations of colors and shapes indicate the three independent experiments performed. Each small dot in the graph corresponds to a specific data point representing an analyzed image or cells. The larger dots represent the average values calculated from the respective data points. A Repeated Measures ANOVA test was used for the comparison between two groups. A value of $P < 0.05$ was considered significant; * $P<0.05$ (RNA-DNA hybrids P=0.0169 and STING P=0.0221). Error bars report the Standard Error.

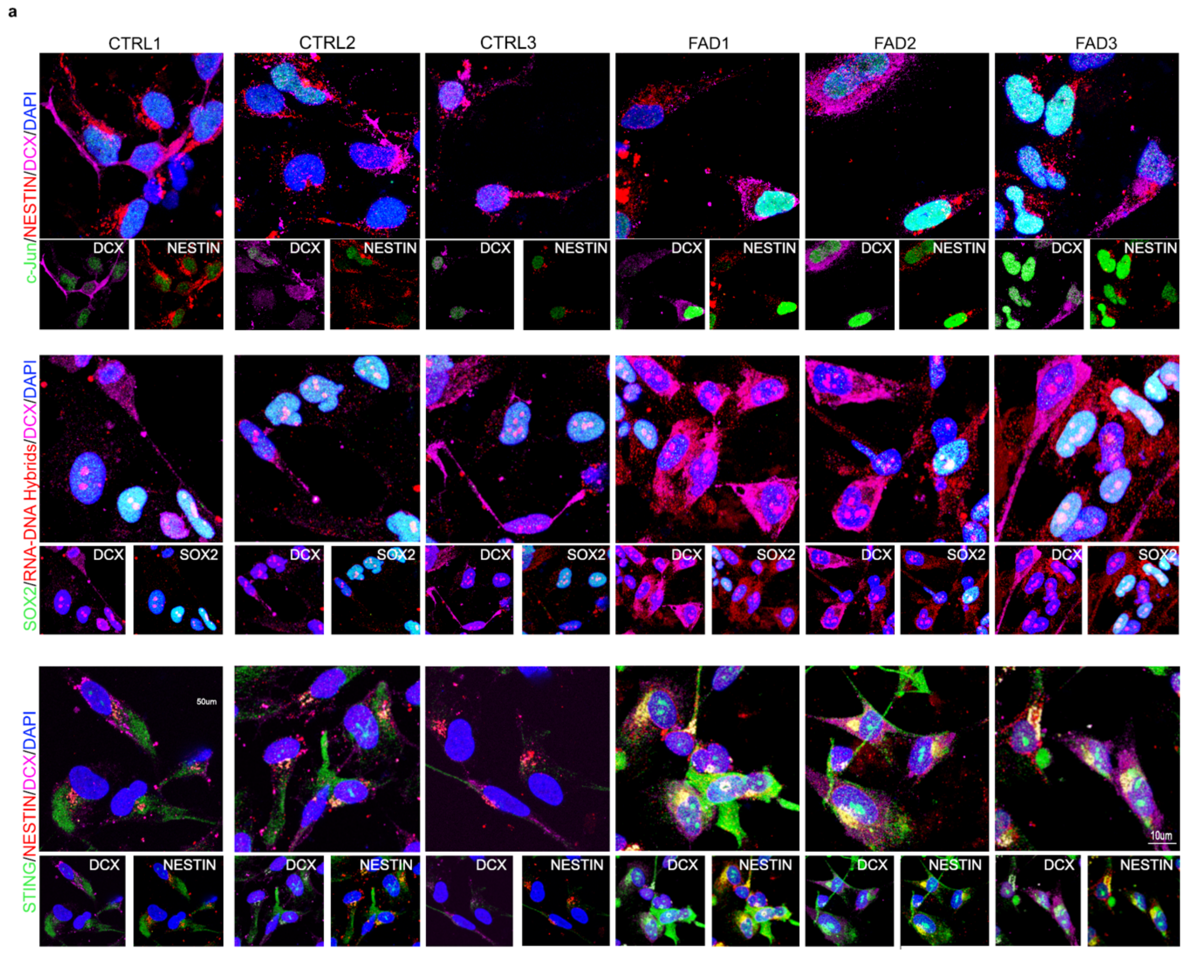

Supplementary Figure 5. **Validation of c-Jun, RNA-DNA hybrids, and cGAS-STING axis in early and late progenitors.** Upper immunofluorescence panel for c-Jun (green), NESTIN, (red) and DCX (magenta). In all FAD progenitors, c-Jun is overexpressed compared to the CTRLs. Central immunofluorescence panel for SOX2 (green), RNA-DNA hybrids (red) and DCX (magenta). All FAD progenitors show a cytoplasmic accumulation of RNA-DNA hybrids compared to the CTRLs. Bottom immunofluorescence panel for STING (green), NESTIN (red) and DCX (magenta). All FAD progenitors show an increased level of STING compared to the CTRLs. Scale bar 10um, 60X magnification, 3x zoom. Each IF was replicated three times with similar results (n= 3 independent experiments).

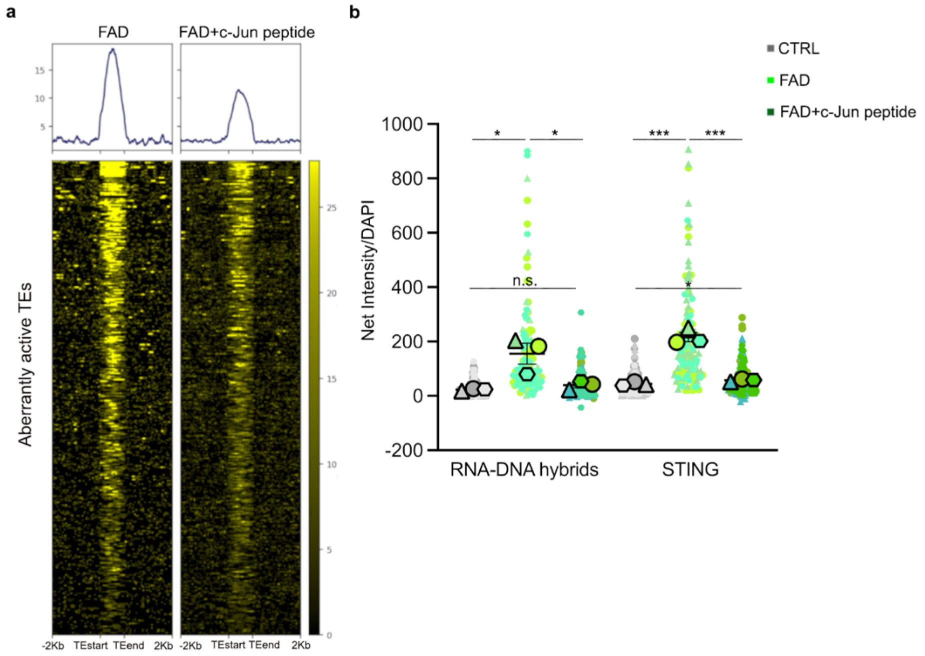

Supplementary Figure 6. **TE activation induces RNA-DNA hybrid accumulation triggering the cGAS-STING cascade and apoptosis in FAD hpNPCs**. **a**. Heatmap showing a reduction in the number of open chromatin regions in FAD+c-Jun peptide at aberrantly active TEs. **b**. Quantification of the immunofluorescences in Figure 7b. The IF data are quantified using a Superplot, which concisely visualizes individual data points and their averages. The distinct combinations of colors and shapes indicate the three independent experiments performed. Each small dot in the graph corresponds to a specific data point representing an analyzed image or cells. The larger dots represent the average values calculated from the respective data points. A Repeated Measures ANOVA test was used for the comparison between two groups. A value of $P < 0.05$ was considered significant; * $P<0.05$ (RNA-DNA hybrids CTRLvsFAD $P=0.0148$; RNA-DNA hybrids FADvsFAD+cJun peptide $P=0.0233$); *** $P<0.001$ (STING CTRLvsFAD $P=4.76e-10$; STING FADvsFAD+cJun peptide $P=3.07e-09$); n.s., not significant (RNA-DNA hybrids CTRLvsFAD+cJun peptide $P=0.0824$). Error bars report the Standard Error.

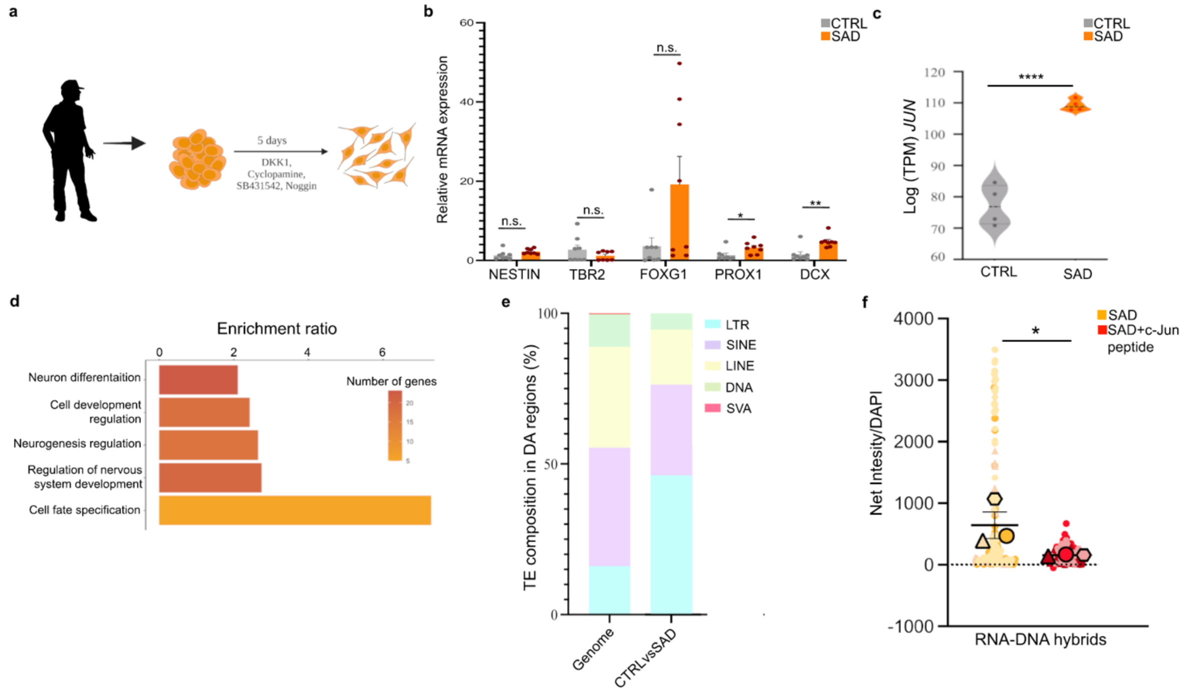

Supplementary Figure 7. **SAD iPSC-derived hippocampal neural progenitors display impaired neurogenesis, gene expression dysregulation, and aberrant activation of TEs**. **a**. The scheme of the protocol for hpNPC differentiation (made with Biorender.com). **b**. qPCR analysis for hpNPC population markers. NESTIN – early precursors; TBR2/FOXG1 – intermediate progenitors; PROX1 – late progenitors; DCX – neuroblasts. Each dot in the bar plot represents an experiment (n= 8 independent experiments). A Two-sided T-test was used for the comparison between two groups. A value of P < 0.05 was considered significant; *P<0.05 (PROX1 P= 0.023463); ** P<0.01 (DCX P= 0.001844); n.s., not signifucant (NESTIN P= 0.057536; TBR2 P=0.233377; FOXG1 P= 0.052947). Error bars report the Standard Error. **c**. Violin plot of log_2(TMP) for *JUN* in SAD hpNPCs compared to CTRLs. **d**. Pathways enriched in the 189 differentially expressed genes (SAD vs CTRL hpNPCs) predicted by WebGestalt. 5% False Discovery Rate (FDR) was used to correct for multiple testing **e**. TE family distribution of the TEs aberrantly de-repressed in SAD progenitors shows an enrichment for LTRs. **f**. Quantification of the immunofluorescences in Figure 7a. The IF data are quantified using a Superplot, which concisely visualizes individual data points and their averages. The distinct combinations of colors and shapes indicate the three independent experiments performed. Each small dot in the graph corresponds to a specific data point representing an analyzed image or cells. The larger dots represent the average values calculated from the respective data points. A Repeated Measures ANOVA test was used for the comparison between two groups. A value

of P < 0.05 was considered significant; * P<0.05 (RNA-DNA hybrids P=0.0125). Error bars report the Standard Error.

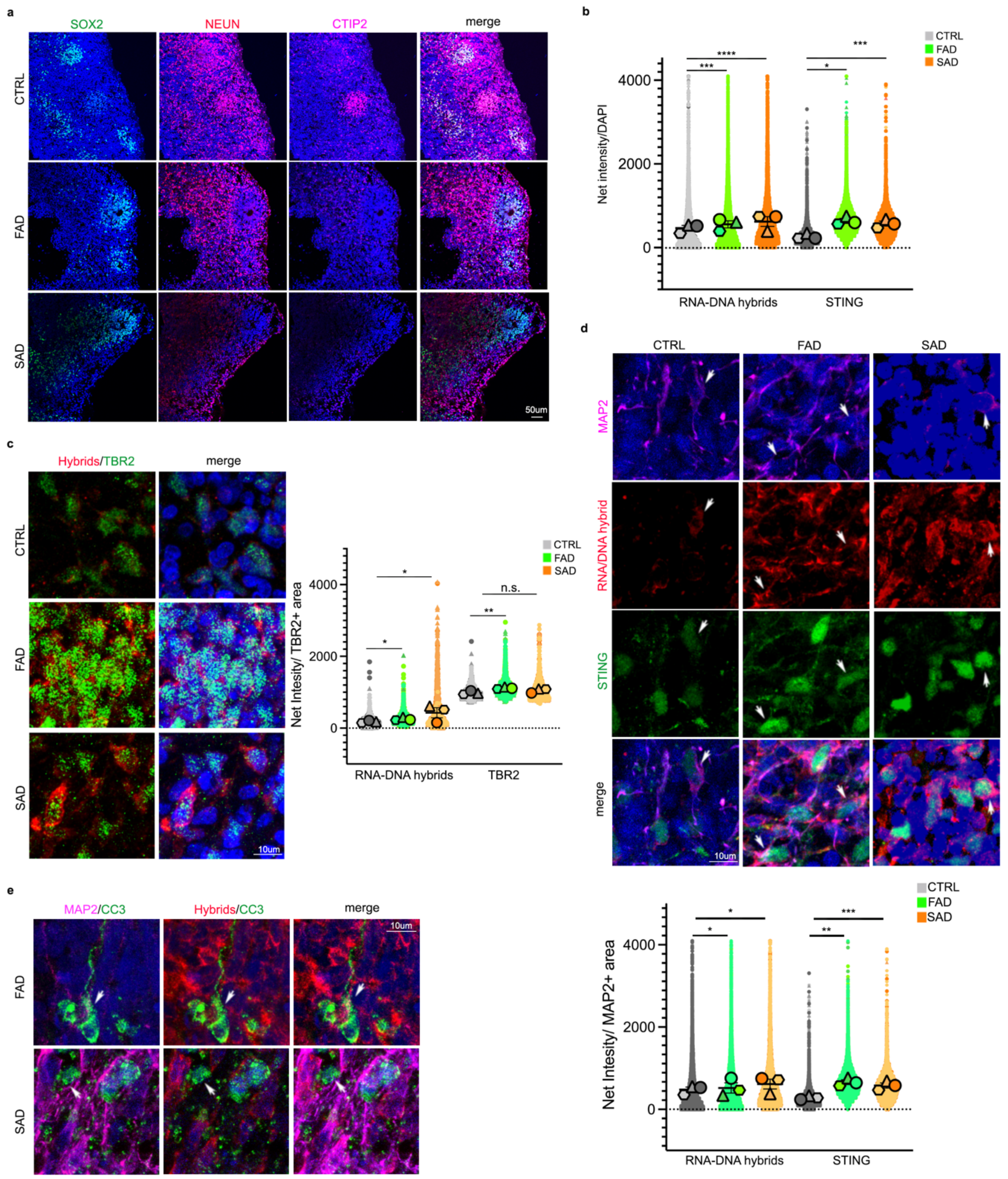

Supplementary Figure 8. **Characterization of TE-derived RNA-DNA hybrids inducing the cGAS-STING cell-death axis in AD cerebral organoids**. **a.** Immunofluorescence for progenitors (SOX2, green signal), immature neurons (NEUN, red signal), and mature neurons (CTIP2, magenta signal) in CTRL and AD (FAD and SAD) organoids. Scale bar 50um, 20X

magnification. DAPI staining on nuclei in blue. Each IF was replicated three times with similar results (n= 3 independent experiments). **b**. Quantification of the immunofluorescences in Figure 7b. The IF data are quantified using a Superplot, which concisely visualizes individual data points and their averages. The distinct combinations of colors and shapes indicate the three independent experiments performed. Each small dot in the graph corresponds to a specific data point representing an analyzed image or cells. The larger dots represent the average values calculated from the respective data points. A Repeated Measures ANOVA test was used for the comparison between two groups. A value of $P < 0.05$ was considered significant; * $P<0.05$ (STING CTRLvsFAD P=0.0123); *** $P<0.001$ (RNA-DNA hybrids CTRLvsFAd P=0.000107; RNA-DNA hybrids CTRLvs SAD P=0.000161; STING CTRLvs SAD P=0.000634). Error bars report the Standard Error. **c**. Immunofluorescence and quantification for intermediate progenitors (TBR2, green signal) and RNA-DNA hybrids (red signal) in CTRL and AD (FAD and SAD) organoids. Scale bar 10um, 60X magnification 4X digital zoom. DAPI staining on nuclei in blue. Each IF was replicated three times with similar results (n= 3 independent experiments). The IF data are quantified using a Superplot, which concisely visualizes individual data points and their averages. The distinct combinations of colors and shapes indicate the three independent experiments performed. Each small dot in the graph corresponds to a specific data point representing an analyzed image or cells. The larger dots represent the average values calculated from the respective data points. A Repeated Measures ANOVA test was used for the comparison between two groups. A value of $P < 0.05$ was considered significant; * $P<0.05$ (RNA-DNA hybrids CTRLvsFAD P=0.0195; RNA-DNA hybrids CTRLvs SAD P=0.0311); ** $P<0.01$ (TBR2+ CTRLvsFAD P=0.00511); n.s., not significant (TBR2+ CTRLvsSAD P=0.116). Error bars report the Standard Error. **d**. Immunofluorescence for neurons (MAP2, magenta signal) and RNA-DNA hybrids (red signal) and STING (green signal) in CTRL and AD (FAD and SAD) organoids. Scale bar 10um, 60X magnification 4X digital zoom. DAPI staining on nuclei in blue. Each IF was replicated three times with similar results (n= 3 independent experiments). The IF data are quantified using a Superplot, which concisely visualizes individual data points and their averages. The distinct combinations of colors and shapes indicate the three independent experiments performed. Each small dot in the graph corresponds to a specific data point representing an analyzed image or cells. The larger dots represent the average values calculated from the respective data points. A Repeated Measures ANOVA test was used for the comparison between two groups. A value of $P < 0.05$ was considered significant; * $P<0.05$ (RNA-DNA hybrids CTRLvsFAD P=0.0479;

RNA-DNA hybrids CTRLvs SAD P=0.0305); ** P<0.01 (STING CTRLvs FAD P=0.00944); *** P<0.001 01 (STING CTRLvs SAD P=0.000576). Error bars report the Standard Error. **e**. Immunofluorescence and quantification for neurons (MAP2, magenta signal), RNA-DNA hybrids (red signal) and cleaved caspase 3 (CC3; green signal) in FAD and SAD organoids. White arrows indicate MAP2/RNA-DNA hybrid/CC3-positive neurons. Scale bar 10um, 60X magnification 4X digital zoom. DAPI staining on nuclei in blue. Each IF was replicated three times with similar results (n= 3 independent experiments).

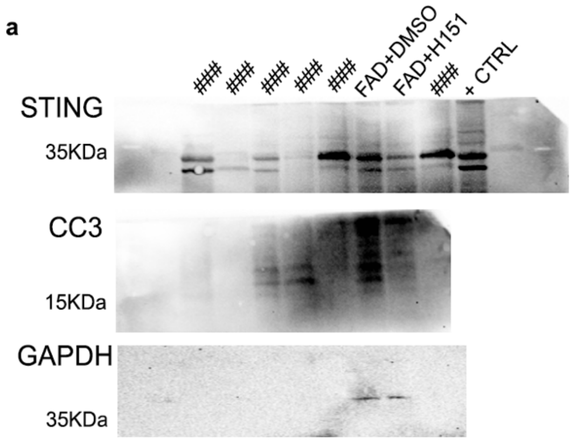

Supplementary Figure 9. **Uncropped scan of Extended Data blots**. **a**. Full uncropped scans of WB in Extended Data Figure 3c.

Supplementary Tables

Supplementary Table1. TMPs values of genes involved in the WNT pathway that are differentially expressed in FAD hpNPCs.

	CTRL	FAD
**DKK1**	3.692171	9.72627767
**DKK3**	57.7179857	223.367699
**JUN**	77.3016	109.701667
**RAC2**	6.636084	51.6482917
**RORA**	2.65834244	7.06845833
**SFRP4**	0.8792335	14.3325863
**WNT2**	0.01490205	3.81743227
**WNT5A**	59.2553505	204.736115
**WNT7A**	0.0380094	0.7898473

Supplementary Table2. List of 63 completely rescued genes after the inhibition of c-Jun phosphorylation.

Genes	p-Value
AC055839.2	0.00325325
ACHE	0.01517861
ALKAL2	0.00151492
ANKRD65	0.01453224
ANKRD7	0.04216957
ARHGAP28	1.16E-05
ATCAY	0.01017104
BACH2	0.04309097
BHLHB9	0.01142904
BMF	0.02976197
CA14	0.01528473
CAPN6	0.04161769
CCNJL	0.00327085
CD200	0.02212538
CDC25A	2.48E-07
CECR2	0.00537765
CLDN10	0.01179058
CNMD	0.00041471
CRABP2	0.01733905
CYP2S1	0.0007133
DACT1	0.0017416
DDAH2	0.02372964
DMRTA1	0.00822087
DOCK8	0.04240049
DTX4	0.00458389
EFEMP1	0.00474706
FAM131B	0.02762477
FBN3	0.00137304
FGF13	0.00034251
FILIP1L	0.00226718
FREM2	0.00891313
GABRB3	0.01588931
GATA2	0.00145519
GLDC	0.00598199
GNG11	0.01356986
GRIK4	0.00287047
HEPH	6.39E-06

Genes	p-Value
HFM1	0.04197935
HSPA2	0.01569638
KANK3	0.02726387
LRRN1	4.83E-08
MAST4	0.00725862
MMP9	9.63E-10
MUC3A	0.03517863
MYCL	0.02064043
NELL1	1.45E-06
NHSL2	0.00028984
NOVA1	0.04902684
PCDHB5	0.00454866
PECAM1	0.00016262
PMEL	0.01091496
PNMA8C	0.01121672
PTPRN	3.41E-07
RASSF5	0.04630868
RCAN2	0.02118692
RNF152	0.00013874
SBK1	0.00063287
SHC2	0.00010682
SLC2A12	0.00026974
SLC6A15	5.49E-05
TMEM130	0.01163338
TMEM178A	3.91E-09
YPEL1	3.72E-05

Supplementary Table 3. Table of antibodies used.

Antibody	Specificity	Host specie	Dilution	Supplier	Reference
OCT4 (MOUSE) Clone 3A2A20 unconjugated	OCT4	mouse	1:200	StemCell Technologies	60093
Human Nanog Antibody	NANOG	goat	1:20	R&D Systems	AF1997
c-Jun Monoclonal Antibody (4H9)	c-Jun	mouse	1:1000 WB	Fisher	MA5-15889
Anti-h nestin AF488 25 ug	NESTIN	mouse	1:200	Invitrogen	5016830
Tbr2 Abcam antibody	TBR2	rabbit	1:200	Abcam	ab216870
Phospho-c-Jun (Ser73) Polyclonal Antibody	Phospho c-Jun	rabbit	1:1000	Fisher	#44-292G
S9.6	RNA-DNA hybrids	mouse	1:500	Kerafast	ENH001

STING Polyclonal Antibody	STING	rabbit	1:100 IF<br>1:1000 WB	ThermoFisher	PA5-23381
Cleaved Caspase-3 (Asp175) (5A1E) Rabbit antibody	Cleaved caspase 3	rabbit	1:200 IF<br>1:1000 WB	CellSignaling	9664T
SCGN Polyclonal Antibody	SCGN	rabbit	1:250	Fisher	PA5-30393
GRIK4 Monoclonal Antibody (8H5G5)	GRIK4	mouse	1:100	Fisher	MA5-31745
Goat Polyclonal Doublecortin antibody	DCX	goat	1:200	Rockland Immunochemicals	600-101-MH8
AB5603 | Anti-Sox2 (rabbit polyclonal)	SOX2	rabbit	1:1000	EMD Millipore	AB5603-100UG
RBFOX3/NeuN Antibody (1B7)	NEUN	mouse	1:1000	Novus Biologicals	NBP1-92693
Mabe1045 | Anti-CTIP2/BCL11B Antibody, clone 25B6	CTIP2	Rat	1:1000	EMD Millipore	MABE1045
Anti-cGAS Antibody	cGAS	rabbit	1:1000	Millipore Sigma	ABF124
MAP2 antibody	MAP2	chicken	1:1000	Novus Biologicals	NB300-213
GAPDH antibody (rabbit) D16H11	GADPH	rabbit	1:1000	Cell Signaling Technologies	5174S
c-Jun (60A8) Rabbit mAb	c-Jun	rabbit	1:200 IF	Cell Signaling Technologies	9165
Horse Anti-mouse IgG, HRP-linked	mouse IgG	horse	1:10000	Cell Signaling Technologies	7076S
Anti-rabbit IgG, HRP-linked	rabbit IgG	goat	1:10000	Cell Signaling Technologies	7074S
Cy™3 AffiniPure Donkey Anti-Rabbit IgG (H+L)	rabbit IgG	Donkey	1:250	Jackson ImmunoResearch	711-165-152
Alexa Fluor® 594 AffiniPure Donkey Anti-Mouse IgG (H+L)	mouse IgG	Donkey	1:500	Jackson ImmunoResearch	715-585-150
Alexa Fluor® 488 AffiniPure Donkey Anti-Rabbit IgG	rabbit IgG	Donkey	1:500	Jackson ImmunoResearch	711-545-152
Alexa Fluor® 488 AffiniPure Donkey Anti-Goat IgG (H+L)	Goat IgG	Donkey	1:500	Jackson ImmunoResearch	705-545-003
Alexa Fluor® 647 AffiniPure Donkey Anti-Mouse IgG (H+L)	mouse IgG	Donkey	1:500	Jackson ImmunoResearch	715-605-150
Goat anti-chicken IgY (H+T) Alexa Flour Plus 647	Chicken IgG	Goat	1:1000	Invitrogen	A32933

Alexa Fluor® 647 AffiniPure Donkey Anti-Rat IgG (H+L)	Rat IgG	Donkey	1:500	Jackson ImmunoResearch	712-605-150

Supplementary Table 4. List of primers used for qRT-PCR analysis.

Primer name	sequence
HERVK env fwd	GCTGCCCTGCCAAACCTGAG
HERVK env rev	CCTGAGTGACATCCCGCTTACC
HERVK gag fwd	AAATAAGACCCAACCGCCAGTAGC
HERVK gag rev	GAATTGCCATGCCTCAGTATCTCC
HERVK pro fwd	GCCGATGAAAAAGCCCGTAAGG
HERVK pro rev	TTGACACTCAGGATTGGCGTTTTC
LTR5HS fwd	GGGCAGCAATACTGCTTTGT
LTR5HS rev	CAATAGTGGGGAGAGGGTCA
L1 ORF1 fwd	CTCGGCAGAAACCCTACAAG
L1 ORF1 rev	CCATGTTTAGCGCTTCCTTC
LH1 fwd	AAAGACACATGCACWCRTATGTT
LH2 rev	TTTCTCAYTYATAGGTGGGA
LH2 fwd	CATGGAATAYTATGCAGCCATAAA
LH3 rev	TCCCACCTATRARTGAGAA
NES fwd	CTTTCAGGACCCCAAGCTGGA
NES rev	CAGGTGTCTCAAGGGTAGCAG
TBR2 fwd	ACCTTCTTCCAGCGTGTGAG
TBR2 rev	TCCTCGTACCTCTTGCTCCT
FOXG1 fwd	AGAAGAACGGCAAGTACGAGA
FOXG1 rev	TGTTGAGGGACAGATTGTGGC
PROX1 fwd	GACTTTGAGGTTCCAGAGAGA
PROX1 rev	TGTAGGCAGTTCGGGGATTTG
DCX fwd	TCAGGGAGTGCGTTACATTTAC
DCX rev	GTTGGGATTGACATTCTTGGTG
18S fwd	ATACATGCCGACGGGCGCTG
18S rev	AGGGGCTGACCGGGTTGGTT
Mitochondrial forward	ACA CCC TCC TAG CCT TAC TAC
Mitochondrial reverse	GAT ATA GGG TCG AAG CCG C
INFG foward	GAGTGTGGAGACCATCAAGGAAG
INFG reverse	TGCTTTGCGTTGGACATTCAAGTC
JUN forward	GCCCAGAGCTAGCGCCTGTG
JUN reverse	CTGTCAACAGCGCCTGGGCA
