# Supplementary material for: JUN upregulation drives aberrant transposable element mobilization, associated innate immune response, and impaired neurogenesis in Alzheimer’s disease
Source: Nat Commun. 2023 Dec 4;14:8021. doi: 10.1038/s41467-023-43728-8 (PMC10696058; doi:10.1038/s41467-023-43728-8)
Supplement: Supplementary file 3 — Reporting Summary [file 41467_2023_43728_MOESM3_ESM.pdf]

nature portfolio

Corresponding author(s): MARCO TRIZZINO
CHIARA SCOPA

Last updated by author(s): 2023/10/24

Reporting Summary

Nature Portfolio wishes to improve the reproducibility of the work that we publish. This form provides structure for consistency and transparency in reporting. For further information on Nature Portfolio policies, see our Editorial Policies and the Editorial Policy Checklist.

Statistics

For all statistical analyses, confirm that the following items are present in the figure legend, table legend, main text, or Methods section.

n/a	Confirmed	
☐	☒	The exact sample size (*n*) for each experimental group/condition, given as a discrete number and unit of measurement
☒	☐	A statement on whether measurements were taken from distinct samples or whether the same sample was measured repeatedly
☐	☒	The statistical test(s) used AND whether they are one- or two-sided<br>*Only common tests should be described solely by name; describe more complex techniques in the Methods section.*
☐	☒	A description of all covariates tested
☐	☒	A description of any assumptions or corrections, such as tests of normality and adjustment for multiple comparisons
☐	☒	A full description of the statistical parameters including central tendency (e.g. means) or other basic estimates (e.g. regression coefficient) AND variation (e.g. standard deviation) or associated estimates of uncertainty (e.g. confidence intervals)
☐	☒	For null hypothesis testing, the test statistic (e.g. *F*, *t*, *r*) with confidence intervals, effect sizes, degrees of freedom and *P* value noted<br>*Give P values as exact values whenever suitable.*
☒	☐	For Bayesian analysis, information on the choice of priors and Markov chain Monte Carlo settings
☒	☐	For hierarchical and complex designs, identification of the appropriate level for tests and full reporting of outcomes
☒	☐	Estimates of effect sizes (e.g. Cohen's *d*, Pearson's *r*), indicating how they were calculated

Our web collection on statistics for biologists contains articles on many of the points above.

Software and code

Policy information about availability of computer code

Data collection	N/A
Data analysis	- Adapter Trimming: TrimGalore! v0.6.5<br>- Read mapping (ATAC-seq and CHIP-seq): BWA mem v.0.7.17<br>- Peak calling (ATAC-seq and ChiP-seq): Macs2 v2.2.71<br>- Gene expression quantification: Kallisto v0.46<br>- Differential Gene Expression: DeSeq2 package<br>- Pathway Analysis: Ingenuity Pathway Analysis suite and webgestalt<br>- Motif Analysis: Meme-ChIP (Meme Suite)<br>- Heatmaps and Average Profiles: Deeptools v3.5.0<br>- Bam file filtering for Q=10 (uniquely mapping reads), sorting, and removal of PCR duplicates: Samtools v1.10

For manuscripts utilizing custom algorithms or software that are central to the research but not yet described in published literature, software must be made available to editors and reviewers. We strongly encourage code deposition in a community repository (e.g. GitHub). See the Nature Portfolio guidelines for submitting code & software for further information.

Data

Policy information about availability of data

All manuscripts must include a data availability statement. This statement should provide the following information, where applicable:

- Accession codes, unique identifiers, or web links for publicly available datasets
- A description of any restrictions on data availability
- For clinical datasets or third party data, please ensure that the statement adheres to our policy

All the Genomic Data (raw and processed) are available on GEO database (GSE213610). The hg19 genome, downloaded from the UCSC Genome Browser, was used for mapping purposes.

Research involving human participants, their data, or biological material

Policy information about studies with human participants or human data. See also policy information about sex, gender (identity/presentation), and sexual orientation and race, ethnicity and racism.

Reporting on sex and gender	N/A
Reporting on race, ethnicity, or other socially relevant groupings	N/A
Population characteristics	N/A
Recruitment	N/A
Ethics oversight	N/A

Note that full information on the approval of the study protocol must also be provided in the manuscript.

Field-specific reporting

Please select the one below that is the best fit for your research. If you are not sure, read the appropriate sections before making your selection.

☒ Life sciences ☐ Behavioural & social sciences ☐ Ecological, evolutionary & environmental sciences

For a reference copy of the document with all sections, see nature.com/documents/nr-reporting-summary-flat.pdf

Life sciences study design

All studies must disclose on these points even when the disclosure is negative.

Sample size	Three conditions: Familial AD, Sporadic AD, and Control. 3 biological replicates per condition (except for the Sporadic AD, for which we only had two lines) and multiple technical replicates per biological replicates. Specifically we used 6 Alzheimer's disease patient iPSC lines (3 familiar alzheimer's disease ones and 2 sporadic Alzheimer's disease ones) and 3 control lines from healthy donor. Two technical replicates per each biological replicate for the NGS experiments: ATAC-seq, RNA-seq three technical replicates for other experiments. The biological replicates always included males and females, of comparable age ranges. One of the Familial AD pair was isogenic. The choses sample size (5 AD lines and 3 CTRL lines) guaranteed sufficient statistical power to replicate the findings biologically at least three times (standard in the field) and detect differentially expressed genes using DESEQ2. It is also worth mentioning that the sample size included ISOGENIC LINES.
Data exclusions	We excluded one FAD replicate from one of the RNA-seq analysis because of poor data quality.
Replication	2 biological replicates per condition: 5 Alzheimer's disease patient iPSC lines (3 familiar alzheimer's disease ones and 2 sporadic Alzheimer's disease ones) vs 3 control lines from healthy donor. Two technical replicates per each biological replicate for the NGS experiments: ATAC-seq, RNA-seq three technical replicates for others experiments. The biological replicates included male and female, comparable age ranges. All the replications were successful.
Randomization	To avoid batch effect, the samples from different conditions (controls, patients) were always processed in the same batch
Blinding	N/A. It was not necessary as we are working with cell lines, that were identified as either Alzheimer or Healthy, and all the cell lines were always processed together in all experiments.

Reporting for specific materials, systems and methods

We require information from authors about some types of materials, experimental systems and methods used in many studies. Here, indicate whether each material, system or method listed is relevant to your study. If you are not sure if a list item applies to your research, read the appropriate section before selecting a response.

Materials & experimental systems

n/a	Involved in the study
☐	☒ Antibodies
☐	☒ Eukaryotic cell lines
☐	☐ Palaeontology and archaeology
☐	☐ Animals and other organisms
☐	☐ Clinical data
☐	☐ Dual use research of concern
☐	☐ Plants

Methods

n/a	Involved in the study
☒	☐ ChIP-seq
☒	☐ Flow cytometry
☒	☐ MRI-based neuroimaging

Antibodies

Antibodies used

OCT4 (MOUSE) Clone 3A2A20 unconjugated (StemCell Technologies, 60093); Human Nanog Antibody (R&D Systems, AF1997); c-Jun Monoclonal Antibody (4H9) (Fisher, MA5-15889); Anti-h nestin AF488 25 ug(Invitrogen, 5016830); Tbr2 abcam antibody (abcam, ab216870); Phospho-c-Jun (Ser73) Polyclonal Antibody (Fisher, #44-292G); S9.6 (kerafast, ENH001);STING Polyclonal Antibody (ThermoFisher, PA5-23381); Cleaved Caspase-3 (Asp175) (5A1E) (CellSignaling, 9664T); SCGN Polyclonal Antibody (FIsher, PA5-30393); GRIK4 Monoclonal Antibody (8H5G5) (Fisher, MA5-31745); Goat Polyclonal Doublecortin antibody (Rockland Immunochemicals, 600-101-MH8); AB5603 | Anti-Sox2 (EMD Millipore, AB5603-100UG); RBFOX3/NeuN Antibody (1B7) (Novus Biologicals, NBP1-92693) Mabe1045 | Anti-CTIP2/BCL11B Antibody, clone 25B6, Anti-CTIP2/BCL11B Antibody, clone 25B6 (EMD Millipore, MABE1045); Anti-cGAS Antibody (Millipore Sigma, ABF124); MAP2 antibody (Novus Biologicals, NB300-213); GAPDH antibody (rabbit) D16H11 (Cell Signaling Technologies, 5174S); c-Jun (60A8) (Cell Signaling Technologies, 9165); Horse Anti-mouse IgG, HRP-linked (Cell Signaling Technologies, 7076S); Anti-rabbit IgG, HRP-linked rabbit IgG (Cell Signaling Technologies, 7074S); Cy™3 AffiniPure Donkey Anti-Rabbit IgG (H+L) (Jackson ImmunoResearch, 711-165-152); Alexa Fluor® 594 AffiniPure Donkey Anti-Mouse IgG (H+L) (Jackson ImmunoResearch,715-585-150); Alexa Fluor® 488 AffiniPure Donkey Anti-Rabbit IgG (Jackson ImmunoResearch, 711-545-152); Alexa Fluor® 488 AffiniPure Donkey Anti-Goat IgG (H+L) (Jackson ImmunoResearch, 705-545-003); Alexa Fluor® 647 AffiniPure Donkey Anti-Mouse IgG (H+L) (Jackson ImmunoResearch,715-605-150); Goat anti-chicken igY (H+T) Alexa Flour Plus 647 (Invitrogen, A32933); Alexa Fluor® 647 AffiniPure Donkey Anti-Rat IgG (H+L) (Jackson ImmunoResearch, 712-605-150).

Validation

OCT4 (MOUSE) Clone 3A2A20 unconjugated (StemCell Technologies, 60093) - This antibody clone has been verified for labeling human ES and iPS cells grown in TeSR™-E8™ (Catalog #05940), mTeSR™1 (Catalog #05850) and TeSR™2 (Catalog #05860). /https://cdn.stemcell.com/media/files/pis/27684-PIS_1_1_1.pdf

Human Nanog Antibody (R&D Systems, AF1997) – Specificity: Detects human Nanog in direct ELISAs and Western blots. Used in over 207 studies. https://www.rndsystems.com/products/human-nanog-antibody_af1997#product-details

c-Jun Monoclonal Antibody (4H9) (Fisher, MA5-15889) - MA5-15889 targets c-Jun in indirect ELISA, IF, IHC, and WB applications and shows reactivity with Human and mouse samples. https://www.thermofisher.com/antibody/product/c-Jun-Antibody-clone-4H9-Monoclonal/MA5-15889. The MA5-15889 immunogen is purified recombinant fragment of human c-Jun expressed in E. Coli.

nti-h nestin AF488 25 ug (Invitrogen, 5016830); The monoclonal antibody 10C2 recognizes human nestin (residues 1464-1614). Nestin is a 220-240 kDa Class VI intermediate filament protein that is expressed in stem cells of the developing nervous system. Expression has also been found in pancreatic islet and mesenchymal precursors in addition to cells with angiogenic potential. Upon differentiation, nestin-containing filaments are replaced by cell type-specific intermediate filament proteins, such as GFAP. Expression has been identified in a variety of cancerous cells including glioblastomas and pancreatic tumors. No cross-reactivity to rodent nestins has been observed using the 10C2 antibody. Applications Reported: This 10C2 antibody has been reported for use in microscopy, immunocytochemistry, immunohistochemical staining, and flow cytometric analysis. Applications Tested: This 10C2 antibody has been tested by immunocytochemistry of formaldehyde-fixed and permeabilized cells. https://www.fishersci.com/shop/products/nestin-monoclonal-antibody-10c2-alexa-fluor-488-ebioscience-invitrogen/5016830

Tbr2 abcam antibody (abcam, ab216870); Suitable for: IHC-P, IHC-Fr, ICC/IF, IPmore details
Unsuitable for: Flow Cyt or WB. Species reactivity: Mouse, Rat, Human. Positive controls for validation: IHC-P: Human tonsil, E14.5 mouse cerebral cortex and E14.5 rat cerebral cortex tissues. IHC-Fr: Mouse E14.5 cerebrum and Rat E14.5 cerebrum tissue. ICC/IF: Mouse primary neuron/glia cells. IP: Mouse E14 brain tissue lysate. Validated in PUBMED: 35794479 AND 34170322. https://www.abcam.com/products/primary-antibodies/tbr2--eomes-antibody-epr21950-241-ab216870.html

Phospho-c-Jun (Ser73) Polyclonal Antibody (Fisher, #44-292G);
The antiserum was produced against a chemically synthesized phosphopeptide derived from the region of human c-Jun that contains serine 73. The sequence is conserved among many species including mouse, rat and chicken. Altered expression of proteins upon cell treatment demonstrates antibody specificity. Western blot analysis of Phospho-c-Jun (Ser73) using Phospho-c-Jun (Ser73) Rabbit Polyclonal Antibody (Product # 44-292G) shows induction of phosphorylation of c-Jun in NIH/3T3 cell line upon PDGF treatment.. https://www.thermofisher.com/antibody/product/Phospho-c-Jun-Ser73-Antibody-Polyclonal/44-292G

S9.6 (kerafast, ENH001) - This mouse monoclonal antibody was generated against a ΦX174 bacteriophage-derived synthetic DNA–RNA antigen and recognizes RNA-DNA hybrids of various lengths. High specificity and affinity for DNA-RNA hybrids. Does NOT cross-react with single-stranded DNA or double-stranded DNA. Used in 94 publications. https://www.kerafast.com/productgroup/432/anti-dna-rna-hybrid-s96-antibody.

STING Polyclonal Antibody (ThermoFisher, PA5-23381) - This Antibody was verified by Knockout to ensure that the antibody binds to the antigen stated. https://www.thermofisher.com/antibody/product/STING-Antibody-Polyclonal/PA5-23381

Cleaved Caspase-3 (Asp175) (5A1E) (CellSignaling, 9664T) - Cleaved Caspase-3 (Asp175) (5A1E) Rabbit mAb detects endogenous levels of the large fragment (17/19 kDa) of activated caspase-3 resulting from cleavage adjacent to Asp175. This antibody does not recognize full-length caspase-3 or other cleaved caspases. Non-specific labeling may be observed by immunofluorescence in specific sub-types of healthy cells in fixed-frozen tissues (e.g. pancreatic alpha-cells). Cytoplasmic background may be observed in human and monkey samples. https://www.cellsignal.com/products/primary-antibodies/cleaved-caspase-3-asp175-5a1e-rabbit-mab/9664
SCGN Polyclonal Antibody (FIsher, PA5-30393) – Validated in PMID: 29727680
GRIK4 Monoclonal Antibody (8H5G5) (Fisher, MA5-31745) – Antibody specificity was tested by the manufacturer with indirect Elisa and with WB using cell lines in which the protein is not expressed. https://www.thermofisher.com/antibody/product/GRIK4-Antibody-clone-8H5G5-Monoclonal/MA5-31745

Goat Polyclonal Doublecortin antibody (Rockland Immunochemicals, 600-101-MH8) - This affinity purified antibody is directed against human Neuronal migration protein doublecortin. This product was affinity purified from monospecific antiserum by immunoaffinity purification. Blast analysis the immunogen sequence shows 100% identity with rat. https://www.rockland.com/categories/primary-antibodies/doublecortin-antibody-600-101-MH8/

Anti-Sox2 (EMD Millipore, AB5603-100UG) – Anti-SOX2 Antibody, Cat. No. AB5603, is a highly specific rabbit polyclonal antibody SOX2 and has been tested for use in Immunocytochemistry, and Immunohistochemistry (Paraffin), and Western Blotting.

RBFOX3/NeuN Antibody (1B7) (Novus Biologicals, NBP1-92693) – Validated in PMID: 30654114 and 23776455. https://www.novusbio.com/products/rbfox3-neun-antibody-1b7_nbp1-92693#datasheet

Anti-CTIP2/BCL11B Antibody, clone 25B6 ¬¬¬– Anti-CTIP2/BCL11B Antibody, clone 25B6, Cat. No. MABE1045, is a highly specific rat monoclonal antibody that targets CTIP2 and has been tested in Immunofluorescence, Immunohistochemistry, and Western Blotting. https://www.merckmillipore.com/GB/en/product/Anti-CTIP2-BCL11B-Antibody-clone-25B6,MM_NF-MABE1045?ReferrerURL=https%3A%2F%2Fwww.google.com%2F#

Anti-cGAS Antibody (Millipore Sigma, ABF124) ¬¬¬– This Anti-cGAS antibody is validated for use in Western Blotting for the detection of cGAS. https://www.merckmillipore.com/GB/en/product/Anti-cGAS-Antibody,MM_NF-ABF124?ReferrerURL=https%3A%2F%2Fwww.google.com%2F#relations

MAP2 antibody (Novus Biologicals, NB300-213) ¬ – This antibody was raised against recombinant constructs of the entire human projection domain, and so recognizes the high molecular MAP2 forms, MAP2A and MAP2B. Knock-down validated. Applications: IF, IHC and WB. Used in 94 publications. https://www.novusbio.com/products/map2-antibody_nb300-213#datasheet

GAPDH antibody (rabbit) D16H11 (Cell Signaling Technologies, 5174S) ¬¬– GAPDH (D16H11) XP® Rabbit mAb detects endogenous levels of total GAPDH protein. Suitable for WB. Used in 6126 publications. https://www.cellsignal.com/products/primary-antibodies/gapdh-d16h11-xp-rabbit-mab/5174?bvstate=pg:2/ct:r
c-Jun (60A8) (Cell Signaling Technologies, 9165) – From the manufacturer: Western blot analysis of extracts from control HeLa cells (lane 1) or c-Jun knockout HeLa cells (lane 2) using c-Jun (60A8) Rabbit mAb #9165. The absence of signal in the c-Jun knockout HeLa cells confirms specificity of the antibody for c-Jun. Used in 1032 publications. https://www.cellsignal.com/products/primary-antibodies/c-jun-60a8-rabbit-mab/9165

Eukaryotic cell lines

Policy information about cell lines and Sex and Gender in Research

Cell line source(s)

Control and Alzheimer's disease iPSC lines were obtained from the Coriell Institute for Medical Research (Camden, NJ).
Control lines: IPSM8Sev3 (male, 65 years old) and iPSM15Sev4 (female, 62 years old).
Familial Alzheimer's disease lines: AG25370 (female, 80 years old) and GM24675 (male, 60 years old).
Sporadic Alzheimer's disease lines: AG27607 (female, 69 years old) and GM24666 (male, 83 years old).
The isogenic iPSC pair FAD3/iso_CTRL3 was obtained by the Jackson Laboratories (revert mutant: JIPSC1054_PSEN2_N141I_REV/WT_human iPSC and Familiar AD mutant: IPSC, JIPSC1052_PSEN2_N141I_SNV/WT_human iPSC). The isogenic pair was generated from the same parental line KOLF2.1J cell line (male).

Authentication

Cell lines were not authenticated for this study.

Mycoplasma contamination

All the cell lines tested negative for mycoplasma contamination.

Commonly misidentified lines
(See ICLAC register)

None of the commonly misidentified cell lines was used.

Palaeontology and Archaeology

Specimen provenance

N/A

Specimen deposition	N/A
Dating methods	N/A

☐ Tick this box to confirm that the raw and calibrated dates are available in the paper or in Supplementary Information.

Ethics oversight	N/A

Note that full information on the approval of the study protocol must also be provided in the manuscript.

Animals and other research organisms

Policy information about studies involving animals; ARRIVE guidelines recommended for reporting animal research, and Sex and Gender in Research

Laboratory animals	N/A
Wild animals	N/A
Reporting on sex	N/A
Field-collected samples	N/A
Ethics oversight	N/A

Note that full information on the approval of the study protocol must also be provided in the manuscript.

Clinical data

Policy information about clinical studies

All manuscripts should comply with the ICMJE guidelines for publication of clinical research and a completed CONSORT checklist must be included with all submissions.

Clinical trial registration	N/A
Study protocol	N/A
Data collection	N/A
Outcomes	N/A

Dual use research of concern

Policy information about dual use research of concern

Hazards

Could the accidental, deliberate or reckless misuse of agents or technologies generated in the work, or the application of information presented in the manuscript, pose a threat to:

No	Yes	
☒	☐	Public health
☒	☐	National security
☒	☐	Crops and/or livestock
☒	☐	Ecosystems
☒	☐	Any other significant area

Experiments of concern

Does the work involve any of these experiments of concern:

No	Yes	
☒	☐	Demonstrate how to render a vaccine ineffective
☒	☐	Confer resistance to therapeutically useful antibiotics or antiviral agents
☒	☐	Enhance the virulence of a pathogen or render a nonpathogen virulent
☒	☐	Increase transmissibility of a pathogen
☒	☐	Alter the host range of a pathogen
☒	☐	Enable evasion of diagnostic/detection modalities
☒	☐	Enable the weaponization of a biological agent or toxin
☒	☐	Any other potentially harmful combination of experiments and agents

Plants

Seed stocks	N/A
Novel plant genotypes	N/A
Authentication	N/A
