# Supplementary figures and images for: JUN upregulation drives aberrant transposable element mobilization, associated innate immune response, and impaired neurogenesis in Alzheimer’s disease
Source: Nat Commun. 2023 Dec 4;14:8021. doi: 10.1038/s41467-023-43728-8 (PMC10696058; doi:10.1038/s41467-023-43728-8)

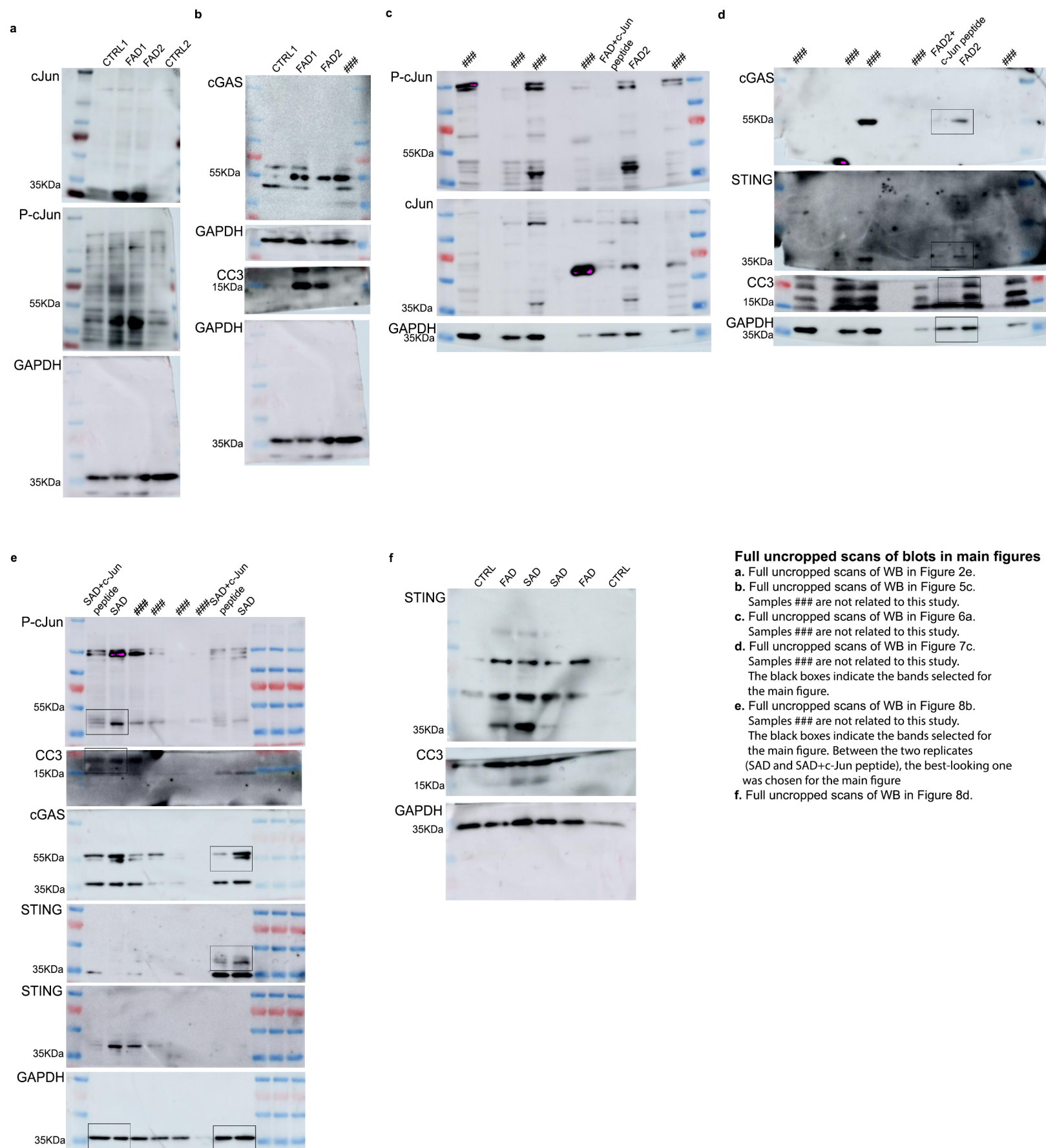

Supplement: Supplementary file 4 — Source Data [file 41467_2023_43728_MOESM4_ESM.zip › Source Data2.pdf]
